# Successful therapeutic rechallenge after a severe episode of high dose methotrexate-induced choreoathetosis: A case report

**DOI:** 10.3892/mco.2019.1898

**Published:** 2019-07-16

**Authors:** Lip Leong Chong, Evelyn Yi Ting Wong, Sheryl Lyn Lucero Santos-Banta, Chee Leong Cheng, Leonard Tan, Eileen Yi Ling Poon, Nagavalli Somasundaram, Mohamad Farid, Tiffany Tang, Miriam Tao, James Boon Kheng Khoo, Vivianne Shih, Daryl Ming Zhe Cheah, Choon Kiat Ong, Soon Thye Lim, Jason Yongsheng Chan

**Affiliations:** 1 Division of Medical Oncology, National Cancer Centre Singapore, Singapore 169610, Republic of Singapore; 2 Department of Anatomical Pathology, Singapore General Hospital, Singapore 169608, Republic of Singapore; 3 SingHealth Duke-NUS Blood Cancer Centre, Singapore 168753, Republic of Singapore; 4 Department of Oncologic Imaging, National Cancer Centre, Singapore 169610, Republic of Singapore; 5 Duke-NUS Medical School, Singapore 169857, Republic of Singapore; 6 Department of Pharmacy, National Cancer Centre Singapore, Singapore 169610, Republic of Singapore; 7 Lymphoma Genomic Translational Research Laboratory, Division of Cellular and Molecular Research, National Cancer Centre Singapore, Singapore 169610, Republic of Singapore; 8 Genome Institute of Singapore, A*STAR, Singapore 138672, Republic of Singapore; 9 Cancer Science Institute of Singapore, National University of Singapore, Singapore 117599, Republic of Singapore

**Keywords:** methotrexate, neurotoxicity, choreoathetosis, leukoencephalopathy

## Abstract

Methotrexate (MTX) is an essential chemotherapy drug used in the treatment of malignancies, but it is known to cause complications to the central nervous system. We report a case of severe MTX neurotoxicity in an adult presenting with choreoathetosis despite a normal clearance of MTX. High dose-MTX has been successfully rechallenged without any neurological sequelae. We reviewed the relevant literature of similar manifestations and summarized their clinical data, magnetic resonance imaging features and treatment given. None of them has recurrence of neurotoxicity. We concluded that it is safe to persist with MTX even after a previous episode of toxic leukoencephalopathy.

## Introduction

Methotrexate is a cell-cycle specific agent which disrupts the metabolism of folic acid and DNA synthesis by inhibiting the enzyme dihydrofolate reductase. It is a commonly used antimetabolite in the treatment of adult and pediatric cancers, including lymphomas, acute lymphoblastic leukemias and osteosarcomas (1). The incidence of acute MTX neurotoxicity is reportedly 3–10% and it depends on the dose, frequency and route of administration of MTX as well as the prophylactic use of leucovorin (2). Acute encephalopathy usually develops within 5 to 14 days after HD MTX, commonly presenting as nausea, headache, seizures, altered mental status or stroke-like symptoms. Choreoathetosis is an unusual and severe presentation of MTX neurotoxicity, and to our knowledge, has not been reported in adults (3,4).

## Case report

A 46 year old woman who presented with confusion and lethargy of 3 weeks duration was diagnosed with an isolated relapse of lymphoma involving the central nervous system (CNS). She had a history of diffuse large B-cell lymphoma (DLBCL) of the breast 12 years ago and was treated then with six cycles of R-CHOP chemotherapy (which included rituximab, cyclophosphamide, doxorubicin, vincristine and prednisolone) and localized radiotherapy to the breast. She did not receive CNS prophylaxis at initial diagnosis. She remained in clinical remission for ten years until she relapsed in the lumbosacral plexus. She received salvage chemotherapy with 4 cycles of R-ESHAP (rituximab, etoposide, methylprednisolone, cytarabine and cisplatin) and went into complete remission.

**Figure 1.**
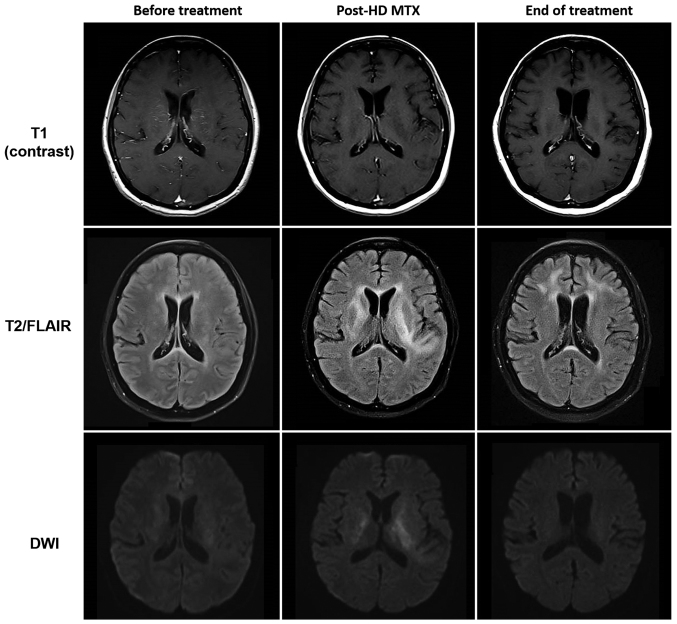
Reversible methotrexate leukoencephalopathy in 46-year-old female patient who presented with seizure, choreoathetosis and altered mental status. HD MTX, high-dose methotrexate; FLAIR, fluid-attenuated inversion recovery; DWI, diffusion-weighted imaging.

**Figure 2.**
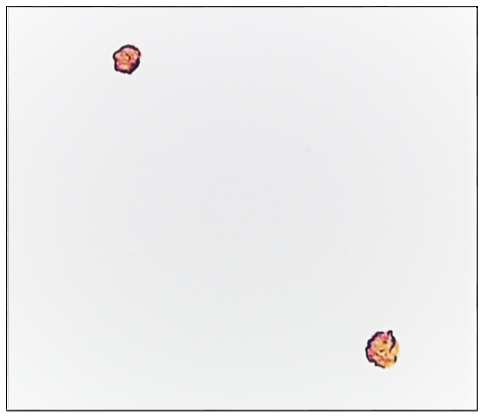
Histopathological analysis of the cerebrospinal fluid. Immunohistochemistry stains with CD20 confirmed the presence of CD20-positive lymphomatous large cells (magnification, ×40).

At the current presentation, gadolinium-enhanced magnetic resonance imaging (MRI) of the brain revealed subcortical white matter hyperintensities on fluid-attenuated inversion recovery (FLAIR) sequence with associated vascular and leptomeningeal enhancement consistent with lymphoma recurrence (Fig. 1). Diagnostic lumbar puncture confirmed the presence of CD20-positive lymphomatous large B cells, typical of leptomeningeal disease (Fig. 2). These large cells also intensely co-expressed CD79a with a Ki-67 proliferation fraction in the region of 60–70% on immunohistochemistry. Flow cytometry analysis was negative for clonal B lymphocytic cells however, this might have been a false negative result due to either limited CSF sample or rapid degeneration of viable lymphocytes. There was no scintigraphic evidence of lymphoma recurrence elsewhere on whole body 18F-FDG PET/CT scan.

**Figure 3.**
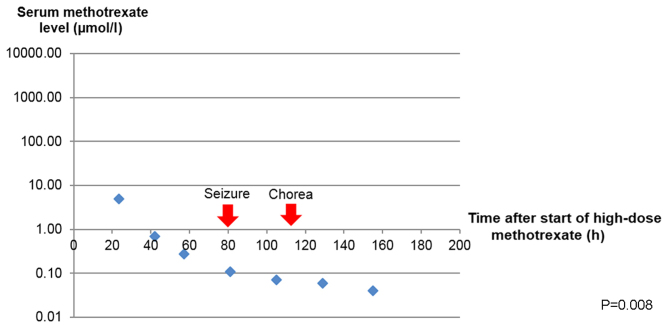
Serum methotrexate level and onset of neurological manifestations. A Friedman test comparing methotrexate level with the baseline was found to be statistically significant, χ^2^(1)=7.0, P=0.008.

She was commenced on dexamethasone, intravenous rituximab and HD MTX infusion at 3.5 g/m^2^ given over 3 h based on Shah's protocol for treatment of newly-diagnosed primary CNS lymphoma (5). IV folinic acid 30 mg 6-hourly was given 24 h after the start of HD MTX infusion until MTX levels <0.05 µmol/l had been achieved (Fig. 3). There was no incidence of toxicity observed on serial monitoring of plasma MTX concentrations. The decrease in serum methotrexate level with time after the start of HD MTX, compared to the baseline methotrexate level was analyzed with Friedman test. All statistical analyses were performed using SPSS17.0 software. A P-value <0.05 (two-tailed) was considered to be of statistical significance.

Four days after HD MTX infusion, she developed sudden jerking movements of the upper limbs with drooling of saliva and extensor posturing of the body. She suffered another complex partial seizure with secondary generalization a few hours later, which aborted spontaneously. She did not have any fever, hypoglycaemia or signs of meningism. Neurological examination was otherwise normal. An urgent CT scan of the brain did not identify any new intracranial lesions nor was there any epileptiform activity on an electroencephalogram (EEG) performed the following day. She was subsequently treated with levetiracetam and there had been no recurrence of seizure since.

Two days later, she was observed to have prolonged involuntary and irregular movements involving bilateral upper extremities with intermittent writhing of the neck and trunk, which would resolve when she slept but recurred when she was awake. A repeat MRI of the brain showed extensive T2 and FLAIR hyperintensities with bilateral and symmetrical involvement of the basal ganglia and periventricular white matter, some of which demonstrated restricted diffusion without any associated solid enhancement. Notably, the previously seen vascular and leptomeningeal enhancement have improved. Further investigations to exclude other causes of choreoathetosis were performed: serum ceruloplasmin, thyroid function, creatine kinase and ferritin were normal, anti-streptolysin O titre (ASOT) and autoimmune workup were negative; as well as a peripheral blood film which did not yield any acanthocytes. A neurologist was consulted for her movement disorder and she was started on tetrabenazine 12.5 mg bd with complete resolution of her symptoms thereafter.

She was successfully rechallenged with HD MTX at a lower dose of 2.5 g/m^2^ at week 3 without any recurrence of neurotoxicity, completing a total of 5 biweekly doses. In addition, she received 2 doses of intrathecal MTX 12 mg at weeks 7 and 9 without any complications. MRI brain was repeated after two months which had shown near complete resolution of white matter changes in the basal ganglia while her lymphoma remained in clinical and radiological remission.

## Discussion

We described a case of acute, reversible methotrexate neurotoxicity in an adult patient with lymphoma who was successfully rechallenged with HD MTX. The differential diagnoses of chorea are broad and can be caused by any structural, metabolic, infectious, autoimmune or malignant involvement of the basal ganglia (6). The bilateral and symmetrical involvements of the basal ganglia in addition to the periventricular white matter were severe and directly accounted for the choreoathetosis observed (7). The putamen and globus pallidus were high in metabolic activity due to an abundance of vascular supply, mitochondria and neurotransmitters compared with other regions of the brain (8). Therefore, this was more suggestive of a toxic-metabolic cause of acute leukoencephalopathy rather than a unilateral neoplastic process.

The diagnosis of MTX-induced encephalopathy in our patient was made based on transient symptoms with full recovery, typical radiographic features and exclusion of other causes after an extensive panel of investigations. We reviewed a series of case reports whereby chorea was one of the main presenting symptoms and summarized them in the tables below (Tables I and II). All the previously reported patients were diagnosed with pediatric ALL on intrathecal MTX with or without HD MTX prior to their neurotoxic symptoms (9–11). MRI brain typically showed focal areas of restricted diffusion, particularly in the basal ganglia and centrum semiovale, which disappeared on follow up imaging. These radiographic findings of transient restricted diffusion in the absence of vascular or perfusion changes were consistent with reversible cytotoxic edema of the white matter from acute MTX leukoencephalopathy (12). All of them, except patient number 2 whom had completed treatment, had received further doses of intrathecal or HD MTX subsequently without any recurrence of neurotoxicity.

There are differences in the treatments used in managing acute MTX neurotoxicity, probably because the exact pathophysiology is still not well understood. The plasma MTX level does not correlate with the incidence of acute neurotoxicity and this relationship is not well-established compared to other adverse effects of MTX such as nephrotoxicity (13). Observations from our patient and case reports from Hong Kong have illustrated that there are no instances of delayed elimination of MTX preceding the neurological symptoms (14). It is widely believed that MTX can cause direct toxic damage to the CNS, potentially by the accumulation of adenosine after MTX inhibition on purine synthesis (15). Methylxanthines have therefore been used in some cases based on their ability to displace adenosine from the central receptors (16). We did not employ methylxanthines in the treatment of our patient but we had continued on folinic acid for another week. Importantly, the patient went on to receive both HD MTX and intrathecal MTX successfully.

Prompt recognition of an acute toxic leukoencephalopathy is important in the management of patients presenting with neurological symptoms and known recent exposure to MTX. This would prevent unnecessary investigations and a delay in the diagnosis of acute MTX neurotoxicity. Diffusion-weighted MRI is a useful imaging modality to detect early changes of cytotoxic edema, which are often transient and reversible (17). It is safe to persist with MTX and that a prior episode of MTX-induced neurotoxicity does not preclude its future use as MTX remains an effective drug in the management of CNS lymphoma.

## Figures and Tables

**Table I.** Clinical features of 5 patients with acute methotrexate neurotoxicity and successful outcomes after rechallenge with MTX.

No.	Age (years)	Sex	Diagnosis	Route of MTX prior to event	Time to event (days)	Neurological symptoms	Duration (days)
1	12	Male	ALL	HD and IT MTX	9	Hemiparesis, bilateral weakness, dysphasia, confusion, emotionality and chorea	1
2	7	Female	ALL	HD and IT MTX	8	Hemiparesis, confusion, emotionality and chorea	6
3	6	Female	ALL	IT MTX only	4	Hypotonia in all limbs and chorea	7
4	17	Male	ALL	IT MTX only	Unknown	Nausea, headache, confusion, right hemiparesis and chorea	<10
5^a^	46	Female	DLBCL	HD MTX only	4	Seizure, confusion and chorea	5

a Described in the present case report. ALL, acute lymphoblastic leukemia; DLBCL, diffuse large B cell lymphoma; MTX, methotrexate; HD MTX, high-dose methotrexate; IT MTX, intrathecal methotrexate.

**Table II.** MRI imaging features and successful outcomes of 5 patients after rechallenge with MTX.

Author, year	No.	MRI imaging features at initial presentation	MRI imaging features on follow-up (duration)	Treatment	Subsequent doses of MTX given	Recurrence of neurotoxicity	(Refs.)
Inaba *et al*, 2008	1	Restricted diffusion in the bilateral centrum semiovale, corona radiata and internal capsules	Normal diffusion with persistent T2 and FLAIR signal increase (7 days)	Aminophylline	HD and IT MTX	No	9
Inaba *et al*, 2008	2	Restricted diffusion in the bilateral basal ganglia, putamina and caudate heads	Increased diffusion with residual small left periatrial white matter signal on T2 and FLAIR (5 months)	Aminophylline	None^a^	No	9
				Lorazepam, clonazepam			
				Diphenhydramine			
Necioğlu *et al*, 2009	3	No signal abnormalities	NA	Haloperidol	IT MTX	No	10
Bota and Dafer, 2009	4	Restricted diffusion in the bilateral centrum semiovale	Normal diffusion with persistent T2 and FLAIR signal increase (2 months)	Folinic acid Dexamethasone	IT MTX	No	11
NA	5^b^	Restricted diffusion in the bilateral basal ganglia and periventricular white matter	Normal diffusion with persistent T2 and FLAIR signal increase (2 months)	Folinic acid Tetrabenazine	HD and IT MTX	No	NA

a Completed planned HD MTX and did not require further chemotherapy.

b Described in the present case report. MRI, magnetic resonance imaging; NA, not applicable; MTX, methotrexate; HD MTX, high-dose methotrexate; IT MTX, intrathecal methotrexate.

## Data Availability

The datasets used and/or analyzed during the current study are available from the corresponding author on reasonable request.
